# Splicing Interruption by Intron Variants in *CSNK2B* Causes Poirier–Bienvenu Neurodevelopmental Syndrome: A Focus on Genotype–Phenotype Correlations

**DOI:** 10.3389/fnins.2022.892768

**Published:** 2022-06-14

**Authors:** Wen Zhang, Fanghua Ye, Shimeng Chen, Jing Peng, Nan Pang, Fei Yin

**Affiliations:** ^1^Department of Pediatrics, Xiangya Hospital, Central South University, Changsha, China; ^2^Hunan Intellectual and Developmental Disabilities Research Center, Changsha, China; ^3^Clinical Research Center for Children Neurodevelopmental Disabilities of Hunan Province, Xiangya Hospital, Central South University, Changsha, China

**Keywords:** *CSNK2B*, Poirier–Bienvenu neurodevelopmental syndrome, genotype, phenotype, intron variants

## Abstract

*CSNK2B* has recently been identified as the causative gene for Poirier–Bienvenu neurodevelopmental syndrome (POBINDS). POBINDS is a rare neurodevelopmental disorder characterized by early-onset epilepsy, developmental delay, hypotonia, and dysmorphism. Limited by the scarcity of patients, the genotype–phenotype correlations in POBINDS are still unclear. In the present study, we describe the clinical and genetic characteristics of eight individuals with POBINDS, most of whom suffered developmental delay, generalized epilepsy, and hypotonia. Minigene experiments confirmed that two intron variants (c.367+5G>A and c.367+6T>C) resulted in the skipping of exon 5, leading to a premature termination of mRNA transcription. Combining our data with the available literature, the types of POBINDS-causing variants included missense, nonsense, frameshift, and splicing, but the variant types do not reflect the clinical severity. Reduced casein kinase 2 holoenzyme activity may represent a unifying pathogenesis. We also found that individuals with missense variants in the zinc finger domain had manageable seizures (*p* = 0.009) and milder intellectual disability (*p* = 0.003) than those with missense variants in other domains of *CSNK2B*. This is the first study of genotype–phenotype correlations in POBINDS, drawing attention to the pathogenicity of intron variants and expanding the understanding of neurodevelopmental disorders.

## Introduction

Poirier–Bienvenu neurodevelopmental syndrome [Online Mendelian Inheritance in Man (OMIM) #618732, POBINDS] is a recently described rare autosomal dominant neurodevelopmental disorder characterized by early-onset seizures, developmental delay, hypotonia, and dysmorphism (Ernst et al., 2021). POBINDS is caused by a *de novo* variant in the *CSNK2B* gene (located at 6p21.33), which encodes the casein kinase 2 enzyme β subunit (CK2β). CK2 is a ubiquitous protein serine/threonine kinase, a heterotetrameric enzyme consisting of two catalytic CK2α or CK2α′ subunits and two regulatory β subunits (Niefind et al., 2001). CK2β is a highly conserved regulatory subunit, and excessive synthesis of CK2β will form dimers that disrupt the complete structure and function of CK2 (Graham and Litchfield, 2000; Niefind et al., 2001). In previous studies, CK2 was found to be widespread in the brain (Guerra et al., 1999), and conditional knockout of *CSNK2B* mice induced age-dependent reduction of grip strength and impaired neuromuscular transmission and synaptic gene expression (Eiber et al., 2019). Knockdown of *CSNK2B* in mouse embryonic neural stem cells (NSC) impaired cell differentiation and reduced dendritic length and branching (Yang et al., 2018). These studies suggest a pivotal role for *CSNK2B* in neurodevelopmental disorders. To date, more than 40 individuals with POBINDS have been reported in the literature (Li et al., 2019; Ernst et al., 2021; Yang et al., 2021), but the genotype–phenotype correlations of POBINDS are still unclear.

In this study, we describe the clinical and genetic characteristics of eight *de novo* individuals with POBINDS, most of whom suffered developmental delay, generalized epilepsy, and hypotonia. Two missense, two nonsense, one frameshift, and three intron variants were detected, with only c.58G>T being reported before (Ernst et al., 2021). Minigene experiments confirmed that two intron variants (c.367+5G>A and c.367+6T>C) resulted in skipping of exon 5, leading to premature termination of mRNA transcription. Combining our data with the available literature, missense, nonsense, frameshift, and splicing site variants were found to cause POBINDS. The variant types do not reflect the severity of POBINDS. Reduced CK2 holoenzyme activity may represent a unifying pathogenesis. Missense variants in the zinc finger domain result in manageable seizures (*p* = 0.009) and milder intellectual disability (ID) (*p* = 0.003) than missense in other domains of *CSNK2B*. This is the first study of the genotype–phenotype correlations of *CSNK2B* variants and expands the knowledge of POBINDS.

## Materials and Methods

### Subject

In this study, a total of eight individuals contributed clinical information and genetic data. This study was reviewed and approved by the Ethics Committee of Xiangya Hospital of Central South University following the Helsinki Declaration. The parents or legal guardian of each individual provided informed consent.

### Next-Generation Sequencing and Sanger Sequencing Verification

Trio-whole-exome sequencing was performed on each family in this study and the methods have been described in detail in previous studies (Peng et al., 2018). All variants reported were confirmed with Sanger sequencing, and the interpretation of variants was conducted under the American College of Medical Genetics guidelines (Richards et al., 2015).

### Minigene Construction

Minigene plasmids covering wild-type “*CSNK2B*_WT (exon 4–6)” and mutants “*CSNK2B*_Mu (exon 4–6)” (Mu1: c.367+2T>C, Mu2: c.367+5G>A, and Mu3: c.367+6T>C) were constructed using the pcDNA3.1 vector, respectively. The constructed minigene plasmids were verified by Sanger sequencing.

### Cell Culture and Transfection

HEK293T cells and Hela cells were obtained from the Kunming Cell Bank of the Chinese Academy of Sciences (Kunming, China), and they were cultured in Dulbecco’s Modified Eagle Medium (Hyclone, United States) supplemented with 10% fetal bovine serum (Gibco, United States), 100 U/ml penicillin, and 100 μg/ml streptomycin (Gibco, United States). Cell culture was performed at 37°C in an incubator filled with 5% CO_2_ and 95% air. The minigene plasmids were transfected into HEK293T cells or Hela cells by LipoMax (Sudgen, China) according to the DNA transfection protocol. The cells were analyzed 48 h after transfection.

### Reverse Transcription-Polymerase Chain Reaction and Sequence Analysis

RNA extraction methods have been described in detail in previous studies (Kong et al., 2015). Total RNA was reverse transcribed using Hifair™ 1st Strand cDNA Synthesis SuperMix for PCR (YEASEN, China). PCR was run on the T100 PCR system (Bio-Rad, United States) using PrimerStarMAX DNA Polymerase (TaKaRa Bio, Japan) according to the manufacturer’s protocol. The primers were synthesized by Tsingke Company (Target exon 4 – exon 6: 5′-CTAGAGAACCCACTGCTTAC, 3′-TAGAAGGCACAGTCGAGG). The purified PCR products were separated and purified *via* 2% agarose and sequenced as described above.

### Systematic Literature Search of Reported *CSNK2B* Variants

We reviewed 57 POBINDS individuals combined with our cases and reported cases (PUBMED, Human Gene Mutation Database, ClinVar, and DECIPHER) involving *CSNK2B.* A total of 82 POBINDS-related variants are summarized, and variants without any detailed clinical information were only aggregated in the information summary (Supplementary Table 1, Patient 58 to Patient 82). Chromosomal microdeletion/microduplications were not involved. The annotation of predicted variants effect was carried by combined annotation dependent depletion (CADD).^1^ Based on the way of affecting protein function, we classified the variants into two categories: loss-of-function (LOF, including start loss, frameshift insertion/deletion, nonsense, and splicing variant), non-LOF (including in-frame insertion/deletion and missense). In the non-LOF group, we also analyzed genotype-phenotype associations based on protein domains. MetaDome^2^ was used for bioinformatics mutation tolerance analysis (Wiel et al., 2019).

### Statistical Analyses

Groups were compared using the Chi-square test, Fisher’s exact test was used where 20% or more of the gird in the Chi-square table were expected to count <5. Significant findings (*p* < 0.05) were calculated as the dominance ratio with 95% confidence intervals. Statistical analysis was performed using SPSS (version 18.0, United States) software.

## Results

### Identification of *de novo CSNK2B* Variants

Eight *CSNK2B* (NM_001320.6) variants were detected in eight individuals; all variants were *de novo* and absent in the gnomAD and ClinVar databases. Only one variant (c.58G>T) was reported before (Ernst et al., 2021). Variant types included nonsense (c.58G>T and c.142C>T), frameshift (c.462_465del), missense (c.325T>C and c.497T>G), and intron (c.367+5G>A, c.367+6T>C, and c.292−1G>A) variants; variant information shown in Supplementary Table 1. Sanger sequencing data of these variants in each family is shown in Supplementary Figure 1.

### Clinical Features of the Eight Individuals

Phenotypes are summarized in Table 1. In the present study, three individuals were male and five were female. All individuals (8/8) had varying degrees of seizures as first signs. Five individuals (5/8) had tonic–clonic seizures, and abnormal electroencephalograms (EEGs) were reported in all individuals (8/8). One individual’s (1/8) MRI showed scattered abnormal signals in the parenchyma. Aside from individual 1, the other seven individuals (7/8) had abnormal neurodevelopmental phenotypes ranging from mild to profound ID. Three individuals (3/8) had mild dysmorphism, which manifested as partial restricted abduction and uplift of eyes, bilateral cryptorchidism, small penis, and a small jaw and prominent forehead, respectively. All individuals (8/8) had epilepsy or global developmental delay starting in the first year of life. Six individuals (6/8) had received one anti-epileptic drug (AED), and two individuals (2/8) received two AEDs. Seizures were completely controlled in seven individuals (7/8), and only one individual (1/8) had medical refraction. The AEDs most frequently reported to be helpful were levetiracetam (4/8), valproic acid (2/8), and oxcarbazepine (2/8). During the follow-up, individuals with global developmental delay (7/8) showed satisfactory improvement in motor, language, and cognitive symptoms but were still distinguishable from their healthy peers.

**TABLE 1:** Genotype and phenotype details for individuals with *CSNK2B* variants.

Patient	Sex/age	Variant, NM_001320.6 (variant type)	First signs/symptoms	Seizure onset/types	AEDs/efficacy	ID	Dysmorphism	Hypotonia	Behavior features	Other features
1	F/3.5 years	c.367+5G>A, (SS)	10 months/seizure	10 months/complex febrile seizure	VPA/seizure free	Normal	Eyes cannot move outwards and upward	−	–	Abnormal EEG
2	F/21 months	c.367+6T>C, (SS)	3 months/seizure	3 months/focal onset, GTC	LEV/seizure free	Mild	–	+	–	Abnormal EEG
3	M/19 months	c.58G>T p.E20* (NS)	3 months/seizure	3 months/GTC	LEV, OXC/seizure free	Moderate	–	+	Introverts, less interaction	Abnormal EEG
4	M/4 years	c.325T>C p.C109R (MS)	8 months/seizure	8 months/GTC	VPA/occasional petit mal	Mild	–	+	Autistic features	Abnormal EEG, MRI at 1 year: abnormal signals scattered in the parenchyma; speech delay, slurred speech
5	F/4 years	c.142C>T p.Q48* (NS)	10 months/seizure	10 months/myoclonic	LEV/seizure free	Mild	–	–	–	Abnormal EEG; motor delay, speak slowly
6	F/30 months	c.462_465del p.Asp155Alafs*70 (FS)	4 months/seizure Delayed neck control	4 months/GTC	LEV, VPA/medically refractory	Profound	–	+	–	Poor head control, poor pursuit of light and objects, cannot sit (6 months). Abnormal EEG
7	M/4 years	c.497T>G p.M166R (MS)	4 months/seizure	4 months/tonic clonic	LEV/seizure free	Moderate	Bilateral cryptorchidism, small penis?	+	–	Abnormal EEG, speech slowly, unable to express long sentences
8	F/5 years	c.292-1G>A, (SS)	4 months/seizure	4 months/focal seizures	OXC/seizure free	Mild	Small jaw, prominent forehead	+	–	Boundary EEG; speech delay; motor delay, motor delay

*, *termination; AED, anti-epileptic drug; EEG, electroencephalogram; FS, frame shift; GTC, generalized tonic–clonic; ID, intellectual disability; LEV, levetiracetam; MRI, magnetic resonance imaging; MS, missense; NS, nonsense; OXC, oxcarbazepine; SS, splice site; VPA, valproic acid.

### Minigene Splicing Assay for Intron Variants (c.367+5G>A and c.367+6T>C)

**FIGURE 1:**
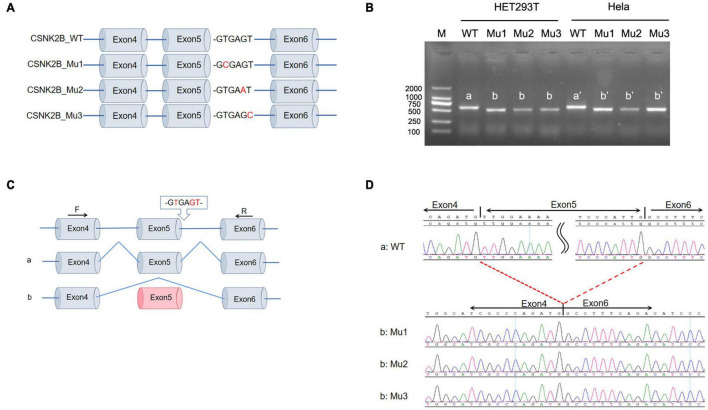
Minigene splicing assay for intron variants. **(A)** Schematic diagram of minigene plasmid construction. Red bases are targeted sites. **(B)** Minigene was transfected into HEK293 cells and Hela cells, respectively. A single band was detected by RT-PCR with a lower molecular weight than wild-type *CSNK2B*. **(C,D)** Sequencing of the above bands revealed that three splicing variants (c.367+2T>C, c.367+5G>A, and c.367+6T>C) all resulted in exon 5 skipping and early termination of the amino acids.

To verify that c.367+5G>A and c.367+6T>C alter the splicing of *CSNK2B*, we performed a minigene assay. We constructed the minigene of *CSNK2B*_WT and *CSNK2B*_Mu (Mu1: c.367+2T>C, Mu2: c.367+5G>A, and Mu3: c.367+6T>C), consisting of exons 4–6 of *CSNK2B* expressed in the pcDNA3.1 vector (Figure 1A). *CSNK2B*_Mu1 was used as a positive control for the minigene assay, which has been confirmed to skip exon 5 in primary cultured fibroblasts (Poirier et al., 2017). Next, we transfected the *CSNK2B* gene into HEK293T and Hela cells, respectively, and reverse transcription-polymerase chain reaction (RT-PCR) results detected a single band of predicted size (Figure 1B). After sequencing, we found that all three splicing variants resulted in skipping of exon 5 (Figures 1C,D). This skipping leads to the insertion of a premature termination codon at p.Leu98Alafs*11, confirmed by RT-PCR (Poirier et al., 2017). These data suggested that these variants resulted in the loss of function of *CSNK2B*.

### Genotype–Phenotype Association Analysis for Reported Cases

**FIGURE 2:**
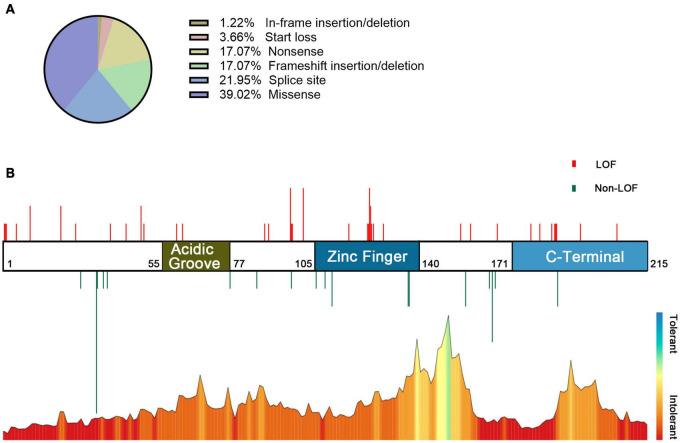
Information on POBINDS-causing CSNK2B mutations (*n* = 82). **(A)** Distribution of mutation types. **(B)** Schematic representation of the mutation distribution (top) and “MetaDome” tolerance level through whole gene (blew). Red vertical lines represent LOF mutations (including start loss, frameshift insertion/deletion, nonsense, and splicing site), and green vertical lines represent non-LOF mutations (including missense and in-frame insertion/deletion). The length of vertical lines represents the number of reported cases. The zinc finger domain is more tolerant than other regions, which is revealed by the “MetaDome” algorithm, this is also consistent with the clinical findings that missense mutation in the zinc finger domain leads to milder phenotypes. References and databases for all sources of mutation information are detailed in Supplementary Table 1.

In the combined analysis of the available literature, we summarized the details of genotypes and phenotypes from 57 cases. Among 57 cases, 48 variants were reported, including 16 missense variants (p.Asp32Asn and p.His165Arg were found in 3 patients and p.Cys137Phe and p.Arg111Pro in 2 patients), 11 splicing variants (including c.558-3T>C, c.367+5G>A, and c.367+6T>C, all confirmed alter splicing), 9 frameshift variants, 8 nonsense variants (c.58G>T, c.139C>T, and c.303C>A were found in two individuals, respectively), 3 start loss variants, and 1 in-frame deletion variant. Phenotypes of *CSNK2B* clinical as Table 2, individuals with the same variant showed varying degrees of phenotypic heterogeneity. A total of 82 POBINDS-related variants are summarized (Figures 2A,B), 25 of them without detailed clinical information are excluded in the phenotype–genotype linkage analysis (details in Supplementary Table 1, Patients 58 to Patient 82). Based on the way they affected protein function, we classified the 57 variants with detailed phenotypes into LOF or non-LOF groups. There was no statistically significant difference in the severity of the POBINDS (epilepsy, refractory epilepsy, moderate to profound ID, dysmorphism, and hypotonia) among mutations of LOF compared to non-LOF, except dysmorphisms (*p* = 0.041) (Supplementary Table 2). Among 23 missense cases, the proportion of refractory epilepsy (Fisher *p* = 0.009) and the severity of ID (Fisher *p* = 0.003) were significantly lower when the mutations were located in the zinc finger region compared to other regions, respectively (Supplementary Table 3). Similar correlations were not found between other domains and phenotypes. This matched the relative tolerance calculated by the “MetaDome” algorithm (Figure 2B). These results suggest that individuals with zinc finger region variants have controllable seizures and a milder degree of ID.

**TABLE 2:** Phenotypes of *CSNK2B* clinical and epilepsy characteristics (*n* = 57).

	All (*n* = 57)	LOF (*n* = 34)	Non-LOF (*n* = 23)
Seizures	51	31	19
Epilepsy	48	31	17
**Onset of epilepsy**			
0–6 months	30	18	12
7–12 months	8	6	2
13–24 months	4	3	1
25–36 months	3	2	1
>36 months	3	2	1
**Epilepsy types**			
Generalized tonic or tonic–clonic seizures	27	17	10
Absence seizures	6	2	4
Myoclonic seizures	14	10	4
Focal onset seizures	9	5	4
Multiple seizure types	18	11	7
Antiepileptic drugs used	38	25	13
Effective epilepsy control	28	18	11
GDD/ID	43	29	20
Motor delay	37	26	16
Speech delay	37	25	17
Moderate to profound	21	14	10
Hypotonia	17	13	8
Dysmorphisms	17	10	13
Problematic behavior	10	8	3

*GDD, global developmental delay; ID, intellectual disability; LOF, loss of function, including start loss, frameshift insertion/deletion, nonsense, and splicing variants; non-LOF, including in-frame insertion/deletion.*

*There was no statistically significant difference in the characteristics of the POBINDS among mutations of LOF compared to non-LOF, except dysmorphisms (details in Supplementary Table 2). References and databases for all mutation information are detailed in Supplementary Table 1.*

## Discussion

*CSNK2B* was first reported by Poirier et al. (2017) in two individuals with early-onset epilepsy, developmental delay, hypotonia, and malformations, and was documented as the causative gene for POBINDS by OMIM in 2020.^3^ A total of 57 individuals with *CSNK2B* mutations have been reported to date (including eight cases in this article), and the largest sample study to date was published in *Epilepsia* by Ernst et al. (2021). The clinical phenotype of POBINDS is complex, with great inter-individual variability and uneven severity of phenotype. Although these studies have expanded the phenotype and genotype spectrum of POBINDS, the genotype–phenotype correlations in POBINDS are still unclear.

Two splicing variants (c.367+5G>A and c.367+6T>C) were located in non-canonical splicing sites. The minigene assays confirmed that these two atypical splice variants lead to skipping of exon 5, resulting in the insertion of a premature stop codon in p.Leu98Alafs*11. A total of 11 splice variants (including three in this article) have been reported in the current literature (11/56), of which six (c.175+2T>G, c.367+2T>C, c.367+5G>A, c.367+6T>C, c.292−2A>T, and c.558−3T>C) have been experimentally confirmed to lead to abnormal splicing of *CSNK2B* (Poirier et al., 2017; Yang et al., 2021). The pathogenicity of atypical splicing site variants is difficult to decipher, and our work confirmed that these variants indeed affect splicing, which is valuable for subsequent research.

Frameshift variants often bring in a premature termination codon, leading to nonsense-mediated decay (NMD) (Shoemaker and Green, 2012). However, among frameshift variants of *CSNK2B*, six out of nine [p.(Met132Leufs*110), p.(Asp155Alafs*71), p.(Leu167Serfs*60), p.(Pro179Tyrfs*49), p.(Asn181Thrfs*46), and p.(Lys208Glnfs*38)], induced a prolonged termination codon downstream of the wild-type termination codon, not upstream. In eukaryotes, mRNAs with these variants may not be directly identified by NMD mechanisms. NMD escape was found in other protein-coding extending like COQ8A, but enzyme activities were significantly reduced (Liu et al., 2014). The functional effects of these six frameshift variants in *CSNK2B* need further verification, but extensive amino acid sequence disruption cannot be ignored. Individuals with these six variants did not show any phenotypic pattern. Another 3 frameshift and 11 nonsense variants were predicted to introduce a stop codon, none of which were in the last exon or the last 50 base pairs of the penultimate exon. In this study, these variants were ranked into LOF variants. Individuals with LOF variants did not show any clear correlations in symptoms, except dysmorphisms (*p* = 0.041). There may be some objective reasons for this phenomenon, such as a small sample, varying phenotype details, and different indicators of developmental assessment in the age strata.

Interestingly, we found some duplicated mutations in the cohort, which may be hotspot mutations or regions. Ernst et al. (2021) and Yang et al. (2021) have reported three start losses with different variants (c.1A>G, c.2T>A, and c.3G>A). The effect of the start loss mutations is still unpredictable. Firstly, the phenotypes of these three individuals are polytropic, c.1A>G showed only mild speech and motor developmental delay. c.2T>A had mild ID, while c.3G>A had profound ID. c.2T>A and c.3G>A were both diagnosed with epilepsy, c.2T>A showed drug-refractory epilepsy, while c.3G>A showed seizure-free. Second, the start loss of *CSNK2B* was also recorded in the gnomAD database^4^ with a relative high population frequencies. Considering that the start loss variant may theoretically affect the open reading frame and leads to gene silencing (Chen et al., 2020), or re-initiate translation by another “ATG” triple nucleotide and mainly affects phenotypic penetrance (Benkirane et al., 2021), whether those variants are pathogenic requires further study. There were eight individuals with c.G94 mutation, and detailed phenotypes were available for four individuals. In these four individuals, some mutual characteristics were found, such as moderate to severe ID, and malformations. Three individuals had seizures and two were diagnosed with epilepsy, both of which manifested as absence epilepsy and drug-refractory. These phenotype profiles suggest that mutations at the c.G94 may be involved in signaling a variety of developmental processes, such as organogenesis (Bandyopadhyay et al., 2016). The untranslated region (UTR) of exon 5 (c.292-367) may be a hotspot region for *CSNK2B* mutations. A total of 11 individuals were reported in this region, 5 individuals with detailed phenotypes available. Four individuals were diagnosed with epilepsy, all with effective antiepileptic treatment. Three with seizures before 6 months of age. The degree of ID varied from mild to severe, except one individual, had normal development (c.367+5G>A), and three cases had malformations (c.292-1G>A, c.367+2T>C, and c.367+5G>A). Although all five splice site mutations have been experimentally confirmed to cause skipping of exon 5 (except for c.292-1G>A), the varied clinical phenotypes suggest that *CSNK2B* may influence POBINDS onset and progression through some unknown pathways.

Regarding the location of the mutation, the individuals with variants in the zinc finger domain (residues 105–140) had manageable seizures and milder ID, which was inextricably linked to the structure and function of CK2β. Zinc finger structures play a crucial role in the regulation of transcription, adaptation of protein interactions, and post-transcriptional regulation in eukaryotes (Laity et al., 2001; Ecco et al., 2017). Zinc finger mutations (cys109, 114) result in the inability of CK2β to form dimers and loss of the interaction with the catalytic subunit CK2α, which is accompanied by increased degradation of the mutant protein (Canton et al., 2001). The individual in our cohort (c.325T>C) was mildly affected and had manageable epilepsy, mild ID, and mainly language delay. This does not appear to be parallel to the severity of the mutation. However, the situation *in vivo* is complex, and developmental delay may be a direct cause of seizures or due to genetic pleiotropy. Increased degradation of CK2β degradation may trigger a compensatory mechanism that regulates CK2β protein levels. Our data reveal corresponding associations from both clinical and bioinformatics analysis, suggesting that the mutation in this region may be less harmful, and also worth further functional study.

In addition to the zinc finger region, CK2β includes the N-terminal (residues 1–54) containing the autophosphorylation site, an acidic groove region (residues 55–77), and a C-terminal (residues 171–215) that stabilizes the CK2β-CK2α interaction (Reed et al., 1994; Bidwai et al., 1995). We compared missense variants across domains, and the results were not convincing due to the small number of variants. The C-terminal-truncated CK2β loses its ability to bind stably to the catalytic subunit (Sarno et al., 2000). p.(Pro179Tyrfs*49)-mutated CK2β is unable to bind to the CK2α, although it is still expressed (Nakashima et al., 2019). The frameshift of *CSNK2B* (c.499del) resulted in decreased protein expression (Yang et al., 2021). These studies suggest that the *CSNK2B* mutation is likely to affect the function of CK2 in a haploinsufficient manner. However, we cannot exclude a dominant-negative effect of CK2β. It was previously shown that although *CSNK2B* heterozygous mice did not differ significantly from wild-type mice in CK2β expression levels, some of the heterozygous mice failed to survive (Blond et al., 2005). This may have been due to the mutated CK2β forming non-functional tetramers that inhibited the normal catalytic activity of the wild-type CK2α. Such phenomena are not uncommon in proteins that perform functions in multimeric forms (Ahn et al., 2003). The reduced CK2 holoenzyme activity may represent a uniform pathogenesis.

The severity of the neurological phenotype ultimately depends on the spatiotemporal distribution and catalytic activity of CK2. The CK2 holoenzyme is a heterotetramer composed of two catalytic subunits (α/α′) and two regulatory subunits (β), which can bind as α2β2, αα′β2, or α′2β2 forms. During early development (embryonic and fetal stages), the mRNA levels of CK2β were significantly higher than those in childhood and adulthood (Yang et al., 2018). CK2 activity was higher in cortical pyramidal neurons and large striatal neurons than in other neuronal populations (Castello et al., 2017). Interestingly, in different brain regions of adult rats, the expression of each subunit of CK2 is different at the transcriptional level and the protein level (Castello et al., 2017). This may form distinguishing CK2 heterotetramers in different brain regions, resulting in different enzymatic activity of CK2.

In addition, CK2 is involved in protein phosphorylation, and these affected proteins/signaling pathways may be the main cause of the progression of neurodevelopmental disorders. CK2 catalyzes the phosphorylation of Kv3.1 and calmodulin (Miller, 2000; Macica and Kaczmarek, 2001), the latter of which directly affects the stability of KCNQ2 channels (Benaim and Villalobo, 2002). CK2 is abundantly expressed in the postsynaptic density (Soto et al., 2004), and inhibition of CK2 blocks *N*-methyl-D-aspartate receptor-dependent synaptic transmission and long-term potentiation (Kimura and Matsuki, 2008). Meanwhile, knockdown of *CSNK2B* not only impaired cell differentiation of mouse embryonic NSC (Yang et al., 2018) but also disrupted the migration of mouse immortalized neuronal GN11 cells (Lettieri et al., 2019). The deletion of *CSNK2B* in embryonic NSC impaired the proliferation of forebrain NSCs and inhibited the differentiation of NSCs into oligodendrocyte precursor cells, resulting in defects in brain development (Huillard et al., 2010). Conditional knockout *CSNK2B* mice showed an age-dependent reduction in grip strength, impaired neuromuscular transmission, and inordinate synaptic gene expression (Eiber et al., 2019). Abnormal emotional behavior, including autistic behavior, irritability, depression, and hyperactivity, may be associated with abnormalities in the CK2-mediated dopamine signaling pathway (Rebholz et al., 2009; DiCarlo and Wallace, 2022). CK2 is also involved in signaling for a variety of developmental processes, such as embryogenesis and organogenesis (Bandyopadhyay et al., 2016). The above CK2 signal involvements underlie the phenotypes of neurodevelopmental disorders such as epilepsy, developmental delay, hypotonia, and dysmorphic disorders.

Limitations of this study include the heterogeneity of the clinical data. When analyzing the reported cases, we relied heavily on the authors’ descriptions of the individuals, and the data lacked a standardized assessment. Especially concerning the judgment of developmental delay, age stratification needs to be introduced to standardize the determination of the degree of developmental delay. The reported age of some patients is too young, and some late-onset neurodevelopmental symptoms need to be determined by long-term follow-up. Additionally, the statistical power of our Chi-square and Fisher’s exact test was generally below 0.8, which may omit some true positive results. This question requires more samples to solve. Moreover, the mechanism by which *CSNK2B* variants cause CK2 dysfunction remains unclear, especially the effect of missense variants on CK2, which requires more experimental models. With the increase of the functional study and refinement of phenotypic profiles, the clarity of genotype–phenotype correlations is expected to be further improved.

In conclusion, this study further enriched the phenotype–genotype profile of POBINDS. Minigene experiments confirmed that two intron variants resulted in skipping exon 5, leading to a premature termination of mRNA transcription. The pathogenicity of intronic variants requires more experiments to decipher. Taken together, our analysis results showed that missense, nonsense, frameshift, and splicing variants were common forms of *CSNK2B* variants, and individuals with variants in the zinc finger domain had manageable seizures and milder ID. The variant types do not reflect the severity of the POBINDS. Determining further genotype–phenotype correlations in POBINDS requires more functional research and refinement of phenotypic profiles.

## Data Availability Statement

The original contributions presented in this study are included in the article/Supplementary Material, further inquiries can be directed to the corresponding authors.

## Ethics Statement

This study was reviewed and approved by the Ethics Committee of Xiangya Hospital of Central South University. Written informed consent was obtained from the individual(s), and minor(s)’ legal guardian/next of kin, for the publication of any potentially identifiable images or data included in this article.

## Author Contributions

FY, WZ, and NP designed the research. WZ, FHY, JP, and NP performed the research. WZ, NP, and FY performed the data analysis. WZ, FHY, NP, and FY wrote the manuscript. All authors read, edited, and approved the manuscript.

## Conflict of Interest

The authors declare that the research was conducted in the absence of any commercial or financial relationships that could be construed as a potential conflict of interest.

## Publisher’s Note

All claims expressed in this article are solely those of the authors and do not necessarily represent those of their affiliated organizations, or those of the publisher, the editors and the reviewers. Any product that may be evaluated in this article, or claim that may be made by its manufacturer, is not guaranteed or endorsed by the publisher.

## Footnotes

1
https://cadd.gs.washington.edu/snv

2
https://stuart.radboudumc.nl/metadome/dashboard

3
https://omim.org

4
www.gnomad-sg.org
