# Supplementary figures and images for: Splicing Interruption by Intron Variants in CSNK2B Causes Poirier–Bienvenu Neurodevelopmental Syndrome: A Focus on Genotype–Phenotype Correlations
Source: Front Neurosci. 2022 Jun 14;16:892768. doi: 10.3389/fnins.2022.892768 (PMC9237577; doi:10.3389/fnins.2022.892768)

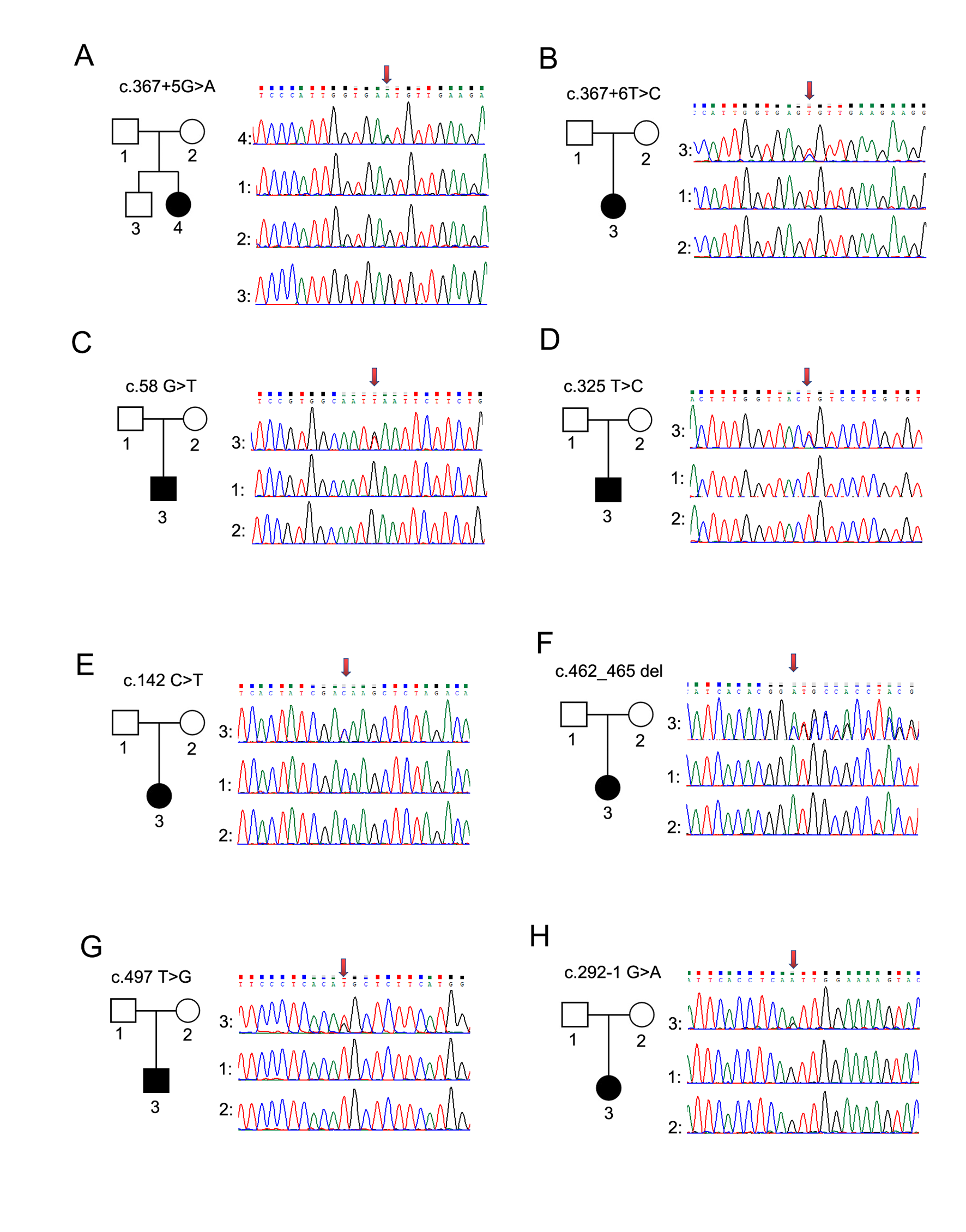

Supplement: Supplementary file 4 [file Image_1.TIF]
